# Effects of Surface Texture and Color on the Visuo-Tactile Perception of Polyurethane Synthetic Leather for Automotive Seats

**DOI:** 10.3390/jemr19030068

**Published:** 2026-06-15

**Authors:** Yuxin Yuan, Shulan Yu, Zhaolong Zhu, Dong Jin, Yu Sun

**Affiliations:** 1 College of Furnishings and Industrial Design, Nanjing Forestry University, Nanjing 210037, China; 1282931323@njfu.edu.cn (Y.Y.); 19557352602@163.com (Y.S.); 2 Co-Innovation Center of Efficient Processing and Utilization of Forest Resources, Nanjing Forestry University, Nanjing 210037, China

**Keywords:** polyurethane synthetic leather, automotive interiors, surface texture, coating color, visuo-tactile perception, eye-tracking

## Abstract

Polyurethane synthetic leather is a widely used covering material in automotive interiors, and its surface coating characteristics directly determine the occupant experience. However, the underlying mechanisms by which these characteristics influence visuo-tactile perception in the context of new energy vehicles (NEVs) require further investigation. In this study, a composite experimental matrix was constructed by combining surface textures with distinct roughness gradients and representative colors extracted via data mining within the HSV color space. Targeting these two surface coating characteristics—color and texture—systematic evaluations were conducted across three independent perception stages: purely visual, purely tactile, and combined visuo-tactile. Eye-tracking metrics, specifically pupil diameter and total fixation duration, were extracted and cross-analyzed alongside multidimensional subjective evaluations. The results indicate that surface texture exerts a significant main effect on both perceived tactile softness and pleasantness, whereas the impact of color variation is remarkably weak. Furthermore, highly complex surface textures lead to prolonged fixation durations, reflecting increased exploratory interest and the high perceptual salience of intricate details rather than mere cognitive workload. Moreover, significant differences in pupil diameter were observed across texture conditions, potentially reflecting the combined influence of low-level image properties and higher-order texture perception. Concurrently, an interference effect of visual features on tactile perception was observed; specifically, the introduction of visual cues (encompassing color and texture) significantly diminished the pleasantness experienced during tactile interaction. These findings elucidate the intrinsic connections between surface coating characteristics and users’ visuo-tactile perception, offering important theoretical guidance and practical implications for optimizing the surface design of automotive polyurethane synthetic leather and enhancing the overall occupant experience.

## 1. Introduction

With the rapid development of electrification and intelligent technologies, the modern automotive cabin is gradually evolving into a “third living space.” In the context of new energy vehicles (NEVs), consumers’ evaluation standards for automotive interiors are progressively extending from basic mechanical properties to multidimensional sensory experiences [1]. The color, material, and finish (CMF) of interior materials can significantly impact users’ sensory interactions, thereby enhancing emotional added value [2]. Translating such subjective material perception into quantifiable engineering parameters is the core objective of Kansei Engineering [3]. Ultimately, multidimensional visual and tactile characteristics directly determine consumers’ aesthetic evaluations and experience preferences [4]. Polyurethane (PU) synthetic leather has been widely applied in automotive seat upholstery [5]. The visuo-tactile experience it provides is fundamentally dominated by micro- and macroscopic topographical features [6]. Advanced embossing processes construct surface textures whose mechanical dimensions significantly affect tactile perception upon human–seat contact [7], acting as core variables that dictate subjective comfort in the cabin [8].

### 1.1. Visuo-Tactile Perception and Crossmodal Integration

Compared to traditional unimodal visual evaluation, material perception relies on both visual and tactile channels, presenting greater challenges [9]. When initial visual expectations conflict with actual tactile feedback, the crossmodal correspondences established in the human brain through long-term experience are easily disrupted [10], consequently triggering measurable perceptual incongruence [11]. Furthermore, a material’s surface texture not only constitutes objective friction but also elicits strong subjective emotional preference responses [12]. In real cabin interactions, vision and touch are usually integrated dynamically and synchronously; humans mathematically integrate multisensory information in a statistically optimal fashion [13]. However, processing complex visual stimuli or uncomfortable tactile feedback significantly increases the visual cognitive processing workload of the nervous system [14], which can directly compromise ultimate sensory pleasantness by disrupting the brain’s processing fluency [15]. Despite this, existing perception research on automotive interiors predominantly focuses on the unimodal visual channel or targets traditional rigid materials like solid wood and metal [16]. Furthermore, occupants’ exploration of interior materials relies heavily on the dynamic integration of multiple channels [17] and dynamic mechanical information gathered during continuous exploratory movements (e.g., lateral finger motion) [18]. Recent studies have demonstrated that the color of an interior space significantly impacts human emotions, physiology, and arousal levels, with strong colors putting the brain into a more excited state and affecting mood and well-being [19]. Indeed, the presence of visual information directly influences the strategies and accuracy of subjects during tactile exploration [20].

### 1.2. Oculomotor Behavior and Cognitive Processing

Traditional consumer preference studies rely heavily on subjective questionnaire scales, which may fail to fully capture individuals’ subconscious cognitive workloads and processing mechanisms [21]. Existing studies demonstrate that incorporating objective physiological metrics can more accurately and unbiasedly capture users’ underlying cognitive processes. For example, Guo et al. [22] found that eye-tracking technology can precisely quantify the allocation of users’ visual attention. To objectively evaluate material perception and the aforementioned cognitive resource consumption, this study employs eye-tracking methodology, which has become a foundational tool in human–computer and multimodal interactions for objectively quantifying visual attention dynamics [23]. Rather than utilizing spatial gaze metrics like scanpath analysis or gaze transitions—which are highly effective for complex visual search tasks involving multiple discrete targets [24,25]—this study focuses on an Area of Interest (AOI) encompassing a single, uniform material stimulus. Therefore, total fixation duration and pupil diameter were selected as the primary oculomotor metrics to capture the localized temporal processing demands of the surface textures.

Fixation duration is frequently used to index visual information processing [26]. While prolonged fixations are often associated with increased cognitive workload and visual decoding difficulty [27], oculomotor behavior is multi-determined. In the context of material perception, longer fixation durations do not exclusively reflect cognitive overload; they can also indicate exploratory interest, uncertainty, novelty, or the higher perceptual salience of intricate textural details (e.g., the complex topographical depth of a litchi pattern) [24,26]. Similarly, pupillometry is a well-established physiological marker for cognitive load and emotional arousal [28,29], and its high temporal sensitivity makes it highly relevant for assessing visual fatigue and autonomic responses during sustained visual or digital tasks [30]. However, absolute pupil diameter is a multi-determined signal. When evaluating PU synthetic leathers of varying colors and textures, pupillary responses must be interpreted cautiously. Beyond task-evoked cognitive responses, pupil size is profoundly governed by a set of physiological visual reflexes: it is highly sensitive to surface luminance and specular reflectances (the pupillary light reflex), actively modulated by the spatial frequency and visual complexity of the observed patterns (pattern-evoked pupillary constriction), and influenced by the near-vision accommodation inherently demanded when inspecting micro-topography [28].

### 1.3. Aims and Hypotheses

The present study aims to investigate the comprehensive effects of PU synthetic leather coating characteristics (three surface textures and four representative colors) on users’ subjective judgments (softness and pleasantness) and oculomotor behavior (fixation duration and pupil diameter) across three distinct interaction stages: pure vision, pure touch, and visuo-tactile coupling.

Based on the theoretical framework discussed above, we propose the following explicit hypotheses:

**H1.**  *Surface texture will exert a stronger main effect on subjective tactile softness and pleasantness than color, with a general preference for smoother textures that offer higher processing fluency in tactile interaction.*

**H2.**  *Fixation duration will be significantly influenced by surface texture complexity. Visually complex textures will elicit longer fixation durations compared to smooth textures, reflecting a combination of increased exploratory interest, high perceptual salience of intricate details, and potential uncertainty during visual decoding.*

**H3.**  *Pupil diameter will vary significantly across different surface textures. This variation may reflect the combined influence of low-level image properties and texture-related perceptual processing.*

**H4.**  *In the visuo-tactile coupling stage, crossmodal interference will occur if initial visual cues conflict with actual tactile feedback, thereby modulating the subjective tactile pleasantness reported in the purely tactile stage.*

## 2. Materials and Methods

### 2.1. Participants

A total of 31 healthy undergraduate and graduate students (12 males, 19 females) were recruited for this study, with a mean age of 22.4 years (SD = 2.1 years). The sample size was determined based on an a priori power analysis using G*Power 3.1 software [31]. Assuming a medium effect size (f = 0.25), a significance level of α = 0.05, and a statistical power of 1 − β = 0.80, the minimum sample size required for repeated measures analysis of variance (RM-ANOVA) was calculated to be 28. Thus, the 31 participants in this study met the statistical requirements. All participants were right-handed (assessed using the Edinburgh Handedness Inventory [32]), possessed normal or corrected-to-normal vision and normal color vision (screened using the Color Blindness Examination Chart published by the People’s Medical Publishing House), and had no history of tactile sensory impairment, severe hand calluses, or neurological diseases. To eliminate the interference of prior experience, none of the subjects had been exposed to the specific synthetic leather samples used in this study prior to the experiment. The research protocol was approved by the Ethics Committee of Nanjing Forestry University. All participants were fully informed of the experimental procedure, signed informed consent forms prior to the experiment, and received corresponding compensation upon completion.

### 2.2. Apparatus and Stimuli

**Figure 1:**
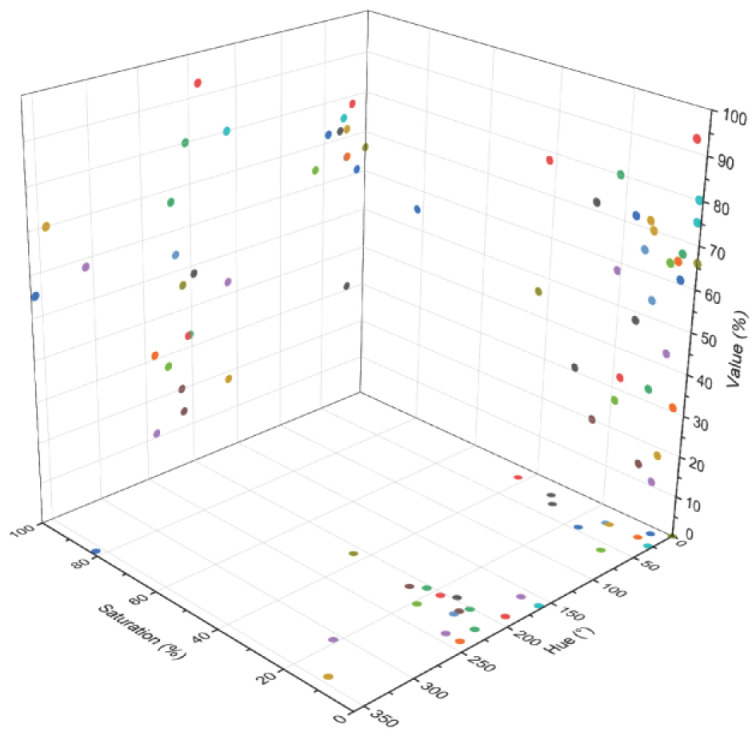
HSV Color Space Visualization. Colors represent the original interior colors extracted from the collected NEV interior images.

To identify representative interior colors for NEVs, 30 interior images of popular models were collected with reference to NEV sales rankings on renowned automotive platforms. Images were processed in Adobe Photoshop to remove irrelevant backgrounds and details, retaining only the dominant color regions of the interiors. Subsequently, utilizing the data-mining-based product color extraction approach proposed by Liu et al. [33], color data were extracted via Adobe Color and mapped onto the HSV color space. As illustrated in Figure 1, the hue distribution of automotive interior colors is relatively broad, whereas saturation primarily concentrates in the low-saturation range of 10–50%, and value concentrates in the medium-to-high range of 50–90%.

Based on this data distribution, to strictly control variables and eliminate extraneous interference from saturation and value differences on subjects’ visuo-tactile perception, four representative hues were selected under conditions of similar saturation (S = 30% ± 10%) and value (V = 70% ± 10%) [31]. Ultimately, four experimental colors were derived: red (H = 355°, S = 35%, V = 70%), brown (H = 45°, S = 30%, V = 75%), green (H = 160°, S = 30%, V = 70%), and blue (H = 210°, S = 35%, V = 75%).

**Figure 2:**
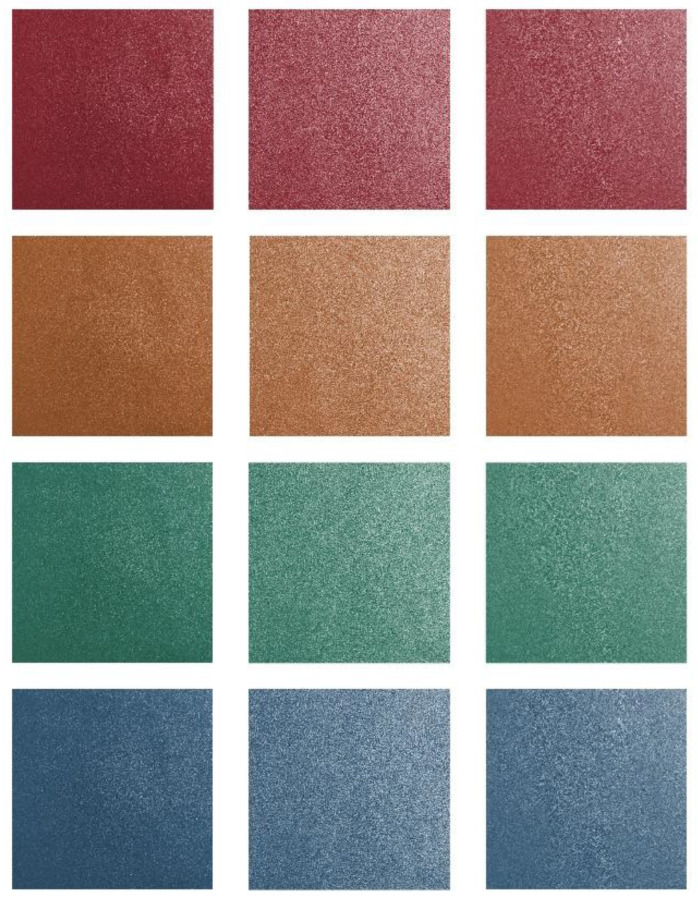
Eye-tracking Experimental Materials. Rows represent different colors (red, brown, green, and blue), and columns represent different texture types (Smooth, Plain, and Litchi).

**Figure 3:**
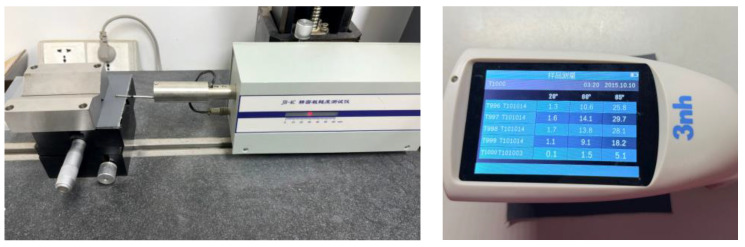
Measurement of objective physical properties.

These four colors were integrated with three typical polyurethane (PU) synthetic leather surface textures (smooth, plain, and litchi), representing low (smooth), medium (plain), and high (litchi) surface roughness gradients based on the objective measurements in Table 1, to fabricate 12 experimental samples, as shown in Figure 2. The sample dimensions were uniformly tailored to 10 cm × 10 cm; this size aligns with the optimal contact area required for “lateral motion” within the Exploratory Procedures (EPs) proposed by Lederman and Klatzky [20], ensuring an accurate assessment of material texture. To quantify surface characteristics, objective characterizations of the samples were conducted according to international standards:

(1) Surface roughness testing: In accordance with the national standard GB/T 1031-2009 [34], a roughness tester (JB-4C, Tarmin Co., Ltd., Shanghai, China) was employed to conduct contact measurements along the sample surface via the stylus method. Each sample was measured at five positions (the center and four corners) and averaged to yield the arithmetical mean roughness (Ra) and the maximum height of the profile (Rz).

(2) Surface gloss (reflectance) determination: Following the national standard for physical and mechanical testing of leather (GB/T 39370-2020) [35], gloss values were recorded at measurement angles of 20°, 60°, and 85° using a gloss meter (3NH-NHG 268, Sanenshi Intelligent Technology Co., Ltd., Guangzhou, Guangdong, China) in a dark room equipped with a cold light source (LED). Each sample was similarly measured at five different positions and averaged. The experimental setups for surface roughness and gloss measurements are shown in Figure 3.

As presented in Table 1, objective measurements verified that the three embossing processes successfully constructed significantly distinct microscopic topographies on the PU coating surfaces. Regarding surface texture, the smooth coating exhibited the lowest roughness parameter (Ra = 3.004 μm), whereas the litchi pattern yielded the highest (Ra = 11.104 μm, Rz = 53.401 μm), indicating the presence of extremely deep microscopic pit structures. Such a complex topographical profile significantly alters the sliding mechanical feedback during subsequent material contact. In terms of surface optical properties, the smooth coating maintained the highest gloss values at both 60° and 85° measurement angles, displaying light source response characteristics inclined toward specular reflection. In contrast, the gloss of the litchi pattern was merely 4.9 GU at the 85° grazing angle, confirming that its deep-pit microstructure induces strong diffuse reflection. These quantified objective physical parameters established a robust materials science baseline for the visuo-tactile stimuli presented to the subjects in this study.

The mean luminance and RMS luminance contrast of all stimulus images were calculated using ImageJ (version 1.54g, National Institutes of Health, Bethesda, MD, USA). All images were converted to 8-bit grayscale, and the mean gray value and standard deviation were measured for each stimulus. The standard deviation was used as an index of RMS luminance contrast. The resulting values are reported in Table 2.

**Figure 4:**
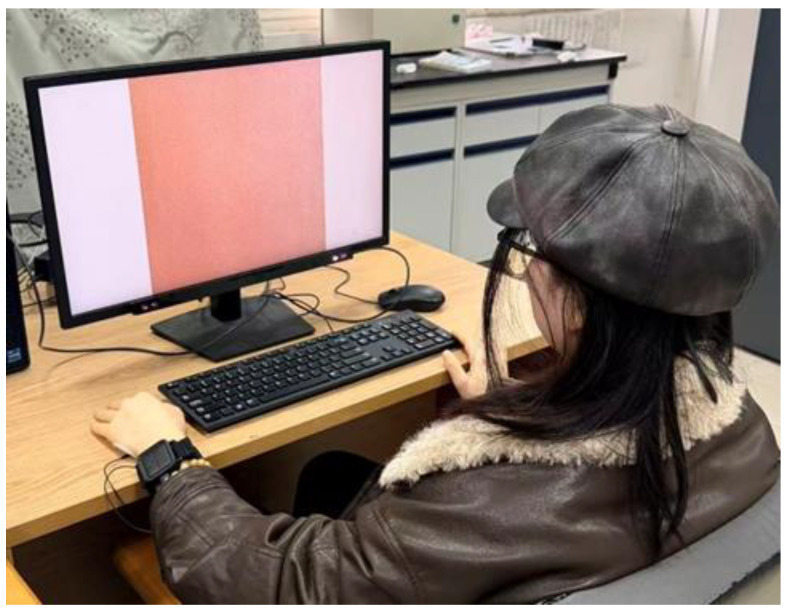
Experimental equipment.

For eye-tracking data acquisition, subjects’ visual attention data were continuously recorded using a Tobii Pro Fusion eye tracker at a sampling rate of 250 Hz. Stimuli were presented on a 23.8-inch LCD monitor with a resolution of 1920 × 1080 pixels. Participants were seated comfortably with their heads unconstrained but instructed to remain still, maintaining a viewing distance of approximately 60 cm. Based on the 10 cm × 10 cm sample dimensions and the viewing distance, each stimulus subtended a visual angle of approximately 9.5°. The eye-tracking experimental setup is illustrated in Figure 4.

Data recording and pre-processing were conducted using the ErgoLAB 3.0 platform (Kingfar International Inc., Beijing, China). Prior to the formal testing, a standard 9-point calibration procedure was performed for each participant, and the experiment proceeded only when the system’s automated validation indicated high tracking accuracy. During the data processing stage, fixation metrics (e.g., total fixation duration) were computed utilizing the software’s built-in Velocity-Threshold Identification (I-VT) algorithm [24], which is the standard filter for classifying fixations and saccades. For pupillometry, the mean pupil diameter was directly extracted using the ErgoLAB system’s default data export module, which automatically handles basic blink artifacts and high-frequency noise removal. Only gaze data with high-confidence validity tags generated by the eye-tracking system were included in the final analysis to ensure data reliability.

### 2.3. Experimental Design and Methods

This study implemented a 3 × 4 full within-subjects design. The core independent variables comprised three specific surface textures and four representative coating colors. To comprehensively evaluate the visuo-tactile perceptual states induced by varying material characteristics, this experiment systematically established dependent variables across two dimensions: subjective experience and objective physiology.

Subjective perceptual measurements:

Three core perceptual dimensions were established, encompassing softness, pleasantness, and visual preference. Softness was measured referring to the tactile perceptual dimensions scale [36] utilizing three items: “hard–soft,” “firm–spongy,” and “rigid–yielding.” Pleasantness was evaluated based on Mehrabian and Russell’s classic emotional model [37] using three items: “happy–unhappy,” “pleased–annoyed,” and “satisfied–dissatisfied.” Visual preference referenced general aesthetic evaluation criteria [38] and was measured with two items: “aesthetic–unaesthetic” and “refined–coarse.” Quantitative scoring was uniformly executed using the 7-point Semantic Differential Scale proposed by Osgood et al. [39], which demonstrates exceptional reliability and validity in Kansei Engineering research. Immediately following the evaluation of each stimulus sample, real-time questionnaire feedback was collected via an electronic platform (SurveyStar) and exported as Excel documents for subsequent statistical analysis.

Softness (1 = extreme negative, 7 = extreme positive): Serving as a core psychophysical dimension of tactile perception, it measures foundational judgments of contact comfort [6,9].

Pleasantness (1 = extreme negative, 7 = extreme positive): Selected based on the study by Etzi et al. [12], it evaluates the sensory emotional valence elicited by the material’s surface texture.

Visual preference (1 = extreme negative, 7 = extreme positive): Referencing consumer preference analysis models [10,15], it gauges the aesthetic inclination toward the material’s CMF appearance.

Objective physiological measurements:

Subjects’ visual attention data were acquired using a Tobii Pro Fusion eye tracker. During the data processing phase, all eye-tracking sequences were uniformly processed within the ErgoLAB_3.0 platform. The specific region containing the presented stimulus sample was uniformly defined as the Area of Interest (AOI). The system established corresponding dwell-time windows for sample validation, from which the total fixation duration and mean pupil diameter were extracted and batch-exported as raw CSV data files. Total fixation duration objectively reflects the sustained period of gaze focus and is widely utilized to characterize the perceptual information decoding difficulty and visual cognitive workload of subjects exploring surface features [16,40]. Furthermore, in a controlled laboratory environment with constant luminance, variations in pupil diameter serve as a highly reliable biological marker for increased visual cognitive engagement and mental workload [41]. The definitions, units, significance, and references for all dependent variables are summarized in Table 3.

### 2.4. Experimental Procedure

**Figure 5:**
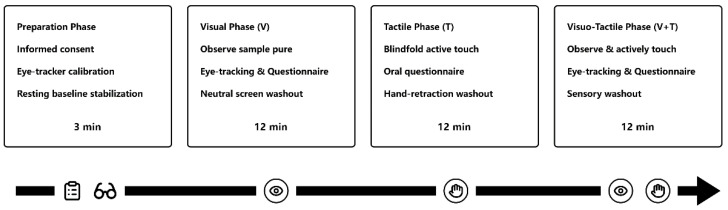
Experimental procedure.

As illustrated in Figure 5, the experiment was conducted in a quiet, well-illuminated, and controlled laboratory environment. The overall procedure was divided into two primary phases: preparation and formal testing.

The preparation phase (approx. 3 min) commenced first: The experimental objectives were explained to the participants, and informed consent was obtained. Subsequently, researchers assisted participants in donning the eye tracker and performing a nine-point calibration. Once stable tracking was confirmed, subjects were instructed to sit quietly and rest to adapt to the laboratory’s luminous environment.

The subsequent formal testing phase consisted of three independent sensory interaction scenarios (i.e., pure vision, pure touch, and combined visuo-tactile). The presentation sequence of all 12 samples within each interaction scenario was completely randomized using a Latin square design to rigorously control for order effects:

Visual scenario (approx. 12 min): Subjects sequentially observed the randomly presented samples without any physical contact. The eye tracker synchronously recorded gaze data. Upon completion of observation, subjects immediately completed the scale scoring for that sample via a mobile device. To prevent superimposition interference from visual afterimages and cumulative cognitive workload, a neutral gray screen was implemented for visual washout after each evaluation, allowing the pupil diameter to return to baseline [28]. A 3 min rest period followed the conclusion of this scenario.

Tactile scenario (approx. 12 min): Subjects wore opaque blindfolds to completely block visual input. With the experimenter’s assistance, subjects utilized their dominant hand (right hand) to perform blind tactile exploration via active lateral motion. Subsequently, they dictated their subjective scale scores, which were logged into the system by the experimenter. After each evaluation, subjects withdrew their hands and executed standardized washout motions to eliminate residual aftereffects and cutaneous receptor fatigue caused by continuous tactile exploration [20]. A 3 min rest period followed this scenario.

Combined visuo-tactile scenario (approx. 12 min): Subjects removed their blindfolds and, maintaining a state of natural eye-hand coordination, simultaneously observed and actively touched the tested samples. The system continuously recorded eye-tracking data during this multimodal processing phase, and scale feedback was collected after each mechanical contact. Comprehensive sensory washout periods were similarly allocated between samples.

The presentation sequence of all 12 samples across all testing stages was completely randomized via a Latin square design to systematically eliminate sequence effects. The total duration for each subject to complete all experimental sessions was approximately 50 min, and appropriate remuneration was awarded upon the conclusion of the experiment.

### 2.5. Data Analysis

Statistical analysis of the experimental data was executed using SPSS 26.0 software. Because the experiment adopted a within-subjects design, a repeated measures analysis of variance (RM-ANOVA) was employed to test the main effects and their interaction effects. Partial eta squared (ηp^2^) was reported as an indicator of effect size to measure the magnitude of variance [42]. When the assumption of sphericity was violated, degrees of freedom were corrected utilizing the Greenhouse-Geisser adjustment [43]. Whenever a main effect or interaction effect achieved statistical significance, simple effect analyses were further conducted, and the *p*-values for post hoc comparisons were rigorously corrected using the Bonferroni method.

## 3. Results

### 3.1. Subjective Perceptual Evaluation

Prior to conducting an in-depth analysis of main and interaction effects, Table 4 summarizes the descriptive statistics (means and standard errors) for the dependent variables across all experimental conditions (texture, color, and interaction stage), including subjective perception (softness, pleasantness) and eye-tracking metrics (pupil diameter, total fixation duration). The subsequent repeated measures analysis of variance (RM-ANOVA) was executed based on these data.

Table 5 presents the RM-ANOVA results for softness and pleasantness. The effects on these two subjective perceptual dimensions are discussed separately below.

#### 3.1.1. Perceived Softness

**Figure 6:**
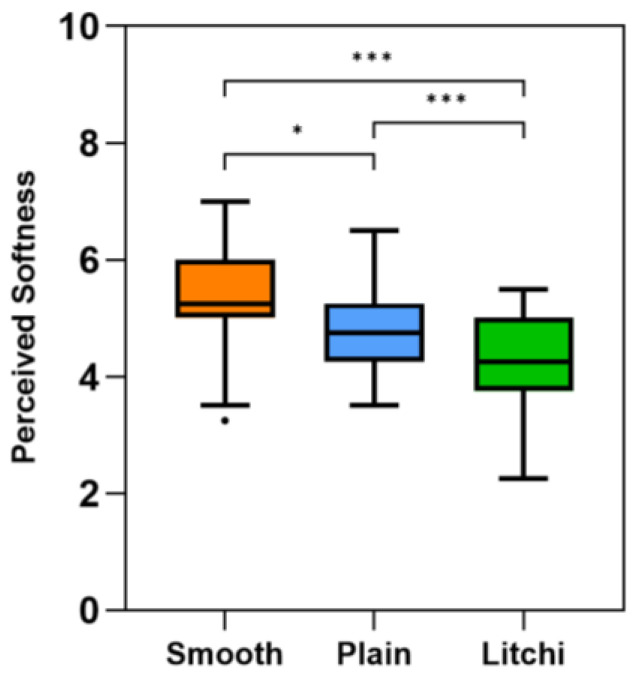
Main effect of surface texture on perceived softness. (* *p* < 0.05, *** *p* < 0.001).

The main effect of surface texture on perceived tactile softness was significant, F = 15.140, *p* < 0.001, ηp^2^ = 0.335, indicating that surface texture significantly influenced perceived softness. The estimated marginal means for softness under each texture condition were: Smooth (M = 5.274, SE = 0.170), Plain (M = 4.823, SE = 0.117), and Litchi (M = 4.242, SE = 0.145). Post hoc pairwise comparisons revealed that the perceived softness of the smooth texture was significantly higher than that of plain (*p* = 0.039) and litchi (*p* = 0.001); concurrently, plain was also significantly softer than litchi (*p* = 0.001). Subjective softness ratings across texture conditions are illustrated in Figure 6.

The main effect of color reached marginal significance, F = 2.728, *p* = 0.049, ηp^2^ = 0.083. However, subsequent multiple comparisons with Bonferroni correction demonstrated no statistically significant difference in softness between any two specific colors (all *p* > 0.05). Furthermore, no significant interaction effect was observed between texture and color (F = 1.029, *p* = 0.408, ηp^2^ = 0.033), suggesting that color did not moderate the impact of surface texture on softness perception.

#### 3.1.2. Perceived Pleasantness

Regarding pleasantness, the main effect of surface texture was significant, F = 4.177, *p* = 0.029, ηp^2^ = 0.122, suggesting that surface texture was associated with differences in pleasantness ratings. The pleasantness scores for respective textures were: Smooth (M = 4.769, SE = 0.151), Plain (M = 4.640, SE = 0.105), and Litchi (M = 4.304, SE = 0.137). However, after applying the Bonferroni correction for multiple comparisons, post hoc tests indicated that the differences in pleasantness between the smooth and litchi coatings (*p* = 0.090), as well as between the plain and litchi coatings (*p* = 0.056), did not reach the strict threshold for statistical significance (*p* > 0.05).

The main effect of color was also significant, F = 3.206, *p* = 0.027, ηp^2^ = 0.097, validating that color variations influenced emotional valance. Pleasantness ratings across color conditions were: Red (M = 4.398, SE = 0.134), Brown (M = 4.638, SE = 0.101), Green (M = 4.534, SE = 0.096), and Blue (M = 4.713, SE = 0.117). Nonetheless, post hoc examinations found no significant differences between specific color pairs (minimum *p* = 0.078). No interaction effect was detected between texture and color (F = 0.849, *p* = 0.561, ηp^2^ = 0.025).

**Figure 7:**
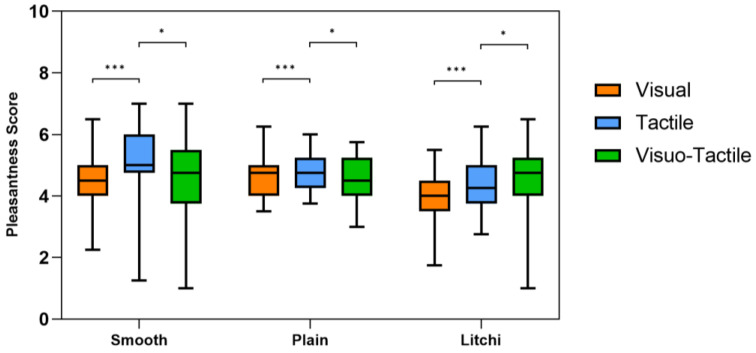
Interaction effects of perception stages and surface textures on pleasantness score. Box plots show the median and interquartile range, with whiskers representing the minimum and maximum values. Brackets and asterisks denote significant differences between perception stages within each surface texture condition (* *p* < 0.05, *** *p* < 0.001).

Notably, the main effect of perceptual stage on pleasantness was highly significant, F = 11.580, *p* < 0.001, ηp^2^ = 0.278. Subjects reported substantially higher pleasantness during the pure tactile stage (M = 4.777, SE = 0.100) compared to the pure visual stage (M = 4.358, SE = 0.099, *p* < 0.001) and the combined visuo-tactile stage (M = 4.578, SE = 0.114, *p* = 0.038). Additionally, a significant interaction between Stage and Texture was identified, F = 2.545, *p* = 0.043, ηp^2^ = 0.078, indicating that the trajectories of pleasantness elicited by different textures varied across sensory modalities, as depicted in Figure 7.

### 3.2. Eye-Tracking Behaviors

Integrating the eye-tracking physiological data, the main effect of surface texture on pupil diameter was highly significant, F(1.62, 48.62) = 131.082, *p* < 0.001, ηp^2^ = 0.814 (Greenhouse-Geisser corrected), indicating systematic differences in pupil responses across texture conditions. Post hoc comparisons revealed highly significant differences among all three textures (Smooth > Plain, *p* < 0.001; Smooth > Litchi, *p* < 0.001; Plain > Litchi, *p* < 0.001).

**Figure 8:**
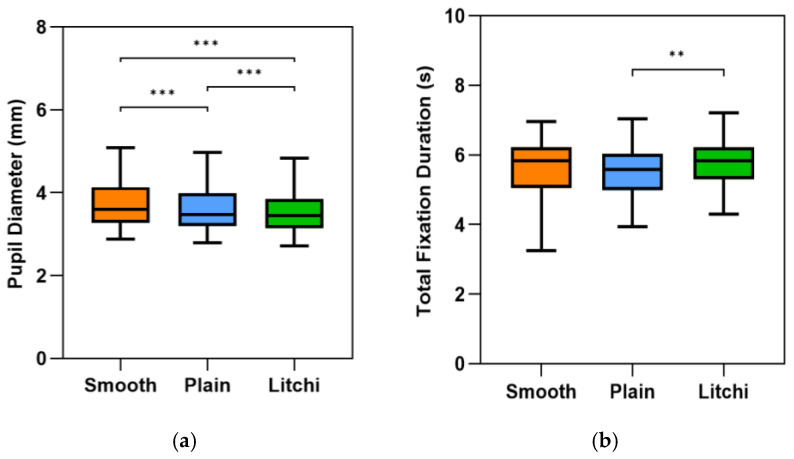
Main effects of surface texture on eye-tracking metrics. (**a**) Mean pupil diameter and (**b**) total fixation duration across different textures. Brackets indicate significant pairwise differences following Bonferroni correction (** *p* < 0.01, *** *p* < 0.001).

Similarly, the main effect of surface texture on total fixation duration was significant, F(1.68, 50.42) = 5.009, *p* = 0.014, ηp^2^ = 0.143 (Greenhouse-Geisser corrected). Post hoc testing demonstrated that the highly complex litchi texture required the longest total fixation duration (M = 5.75 s), which was significantly higher than that of the plain texture (M = 5.51 s, *p* = 0.004). Although the value was numerically greater than that of the smooth texture (M = 5.54 s), it did not reach the threshold of statistical significance (*p* = 0.124). Variations in eye-tracking metrics across texture conditions are presented in Figure 8.

**Figure 9:**
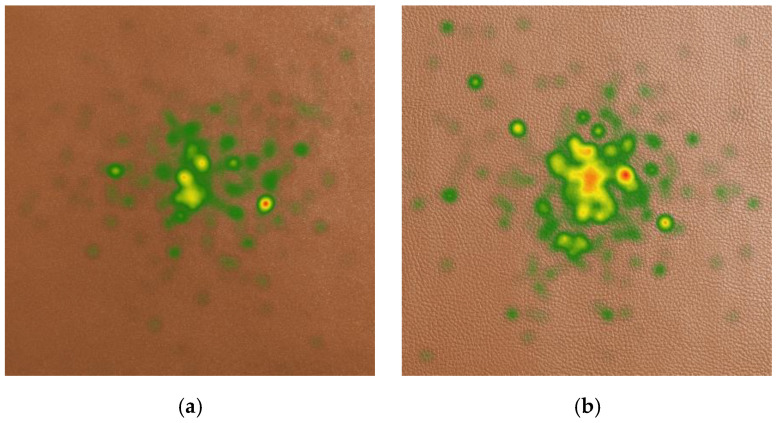
Gaze heatmaps illustrating the spatial distribution of visual attention during the observation of different surface textures. (**a**) Heatmap for the plain/smooth texture, showing relatively concentrated fixations; (**b**) Heatmap for the highly complex litchi texture, displaying a more dispersed and extensive visual exploration pattern. Color intensity indicates fixation density, with green representing lower density, yellow representing medium density, and red representing higher density.

To provide a more intuitive representation of the visual exploration strategies, qualitative gaze heatmaps were generated for the different texture conditions (Figure 9). As shown in the heatmaps, visual attention was relatively concentrated when observing the simple plain texture (Figure 9a). In contrast, the highly complex litchi texture elicited a more dispersed and extensive pattern of fixations (Figure 9b). This distinct spatial distribution of gaze aligns with our quantitative findings on total fixation duration, visually suggesting that more complex surfaces may encourage broader visual exploration.

### 3.3. Correlation Analysis of Subjective and Objective Metrics

Pearson correlation analysis elucidated potential associations between subjective and objective perceptual indicators. In the subjective evaluation dimension, pleasantness exhibited a significant positive correlation with tactile softness (r = 0.569, *p* = 0.001). This demonstrates that, within the subjects’ perceptual framework, enhanced material flexibility during physical contact can directly translate into superior sensory pleasantness. In the objective physiological dimension, total fixation duration—a reflection of visual cognitive workload—showed no significant correlation with subjective visual preference (r = −0.101, *p* = 0.590). Likewise, mean pupil diameter displayed no statistically significant association with visual preference (r = 0.059, *p* = 0.754).

## 4. Discussion

### 4.1. Effects of Surface Texture on Dependent Variables

Consistent with H1, experimental results indicated that surface texture played a significant role in the multimodal visuo-tactile perception of PU synthetic leather, significantly influencing both subjective evaluations and objective eye-tracking behaviors.

Subjectively, participants perceived the smooth coating as providing the significantly highest tactile comfort (softness). While the smooth texture also yielded the highest absolute scores for subjective preference (pleasantness), strict post hoc corrections indicate that specific pairwise differences in pleasantness should be interpreted with caution. Nevertheless, this overall evaluation pattern aligns with the conclusions of Etzi et al. [12], which suggest an inherent human preference for smooth, fine tactile sensations in daily experiences. In the context of actual automotive seat contact, a smooth surface delivers uniform and seamless mechanical feedback, reducing the frictional resistance during finger sliding. This mechanism effectively elevates tactile comfort (softness), which our prior correlation analysis shows is significantly and positively associated with overall subjective pleasantness. This aligns with recent findings in material science, demonstrating that specific surface coating processes can significantly modify a material’s macroscopic roughness and gloss, ultimately determining its interactive performance [44].

Objectively, regarding eye-tracking behavior, our findings supported H2. Subjects exhibited prolonged total fixation durations when confronted with surface textures featuring high visual complexity, such as the litchi pattern. While earlier interpretations sometimes equated longer fixations straightforwardly with higher cognitive workload, fixation behavior is inherently multi-determined [24]. In the context of material perception, the prolonged fixation on the complex litchi pattern should not be interpreted solely as cognitive effort; rather, it may reflect a combination of exploratory interest, novelty, and the perceptual salience of intricate texture details. The macroscopic concave-convex structure of the litchi surface increases visual complexity, which may necessitate longer viewing times for observers to thoroughly explore its physical features. Our eye-tracking results indicated that the variations in surface properties altered participants’ visual scanning behavior. This phenomenon is consistent with findings from other eye-tracking research, which emphasizes that specific visual features (e.g., luminance contrast) [45] and multimodal sensory inputs [46] can strongly direct gaze behavior, reshape attentional distribution, and modify search efficiency.

In accordance with H3, the analysis of pupil diameter revealed systematic variations across different synthetic leather textures. Specifically, the Smooth texture elicited larger pupil diameters, whereas the Litchi texture elicited smaller pupil diameters. These findings indicate that different texture conditions were associated with distinct pupillary responses.

However, as shown in Table 2, the mean luminance and luminance contrast of the stimuli varied across textures, with Litchi stimuli generally exhibiting higher luminance values than Smooth stimuli. Therefore, part of the observed variation in pupil diameter may reflect conventional luminance-driven pupillary responses. This possibility should be considered when interpreting the results.

At the same time, previous studies have demonstrated that pupil dynamics can be influenced not only by low-level physical properties, but also by higher-order visual processing, including the perception of complex spatial patterns and fine structural details [28,46]. Consequently, although the present data do not allow the independent contributions of luminance and texture processing to be fully disentangled, it remains possible that texture-related perceptual processing contributed to the observed pupillary responses. In particular, the more intricate microstructures and surface irregularities of the Litchi texture may have imposed different perceptual processing demands compared with smoother surfaces.

Taken together, these findings support H3 by demonstrating that synthetic leather textures produced significantly different pupillary responses. However, because systematic differences in luminance and contrast were present across texture conditions, the underlying mechanisms should be interpreted with caution. The present results demonstrate a texture-related pupillary effect, but they do not allow definitive conclusions regarding the relative contributions of low-level image properties and higher-level texture perception. Future studies employing luminance-matched stimuli will be necessary to further clarify these contributions.

### 4.2. Effects of Color and Multimodal Interaction Scenarios on Dependent Variables

Concerning the color variable, RM-ANOVA revealed a significant main effect; however, post hoc multiple comparisons failed to identify significant differences between specific hues. This diverges from previous studies that focused solely on two-dimensional pure visual patterns [1]. One possible explanation is that the present study involved visuo-tactile interactions rather than purely visual evaluation. Upon introducing authentic physical tactile feedback, the surface frictional resistance (tactile comfort) transmitted via the skin occupies a substantial proportion in the multisensory weighting allocation [13], thereby relatively masking the visual impact of color. Similar shifts in sensory channel dominance have been documented in studies on other artificial coatings: when surface textures possess robust physical tactile traits, the sensory contribution of coating color to the ultimate subjective preference is noticeably attenuated [4]. This finding offers practical guidance for interior design: during the formulation of synthetic leather CMF solutions, optimizing surface texture (tactile comfort and visual complexity) may take precedence over fine-tuning color parameters.

In the domain of multisensory interaction, as predicted by H4, an intriguing phenomenon was recorded: subjects’ subjective pleasantness ratings during the pure tactile stage were significantly higher than those in the combined visuo-tactile stage. This manifestation may relate to crossmodal interactions or sensory conflict within multimodal integration pathways, consistent with observations in sensory psychology [47,48,49]. During concurrent visuo-tactile evaluation, the expectations generated by the visual appearance of distinct textures might not perfectly align with the actual mechanical feedback obtained upon fingertip contact. While this study did not directly measure the underlying neural integration mechanisms, processing such potential cross-sensory incongruence could lead to divided attention or subtle processing disruptions [11]. This dynamic might subtly interfere with the pure tactile comfort experienced in a vision-deprived state, potentially contributing to a relative decline in overall subjective pleasantness during multimodal evaluation.

### 4.3. Limitations and Future Perspectives

Although this study provided objective physiological insights into the visuo-tactile perception of synthetic leather, certain limitations should be noted to guide future research. First, the participant sample primarily consisted of young university students, which may limit the generalizability of the findings across different age groups or occupational backgrounds. Subsequent research should include participants from a broader demographic to improve external validity. Second, the controlled laboratory environment may not fully capture the complexity of real-world driving conditions. In actual automotive cabins, occupants are exposed to dynamic variables such as transient ambient lighting, vehicle vibration, and divided attention during driving. These contextual factors might influence multisensory interaction strategies differently than in controlled experimental conditions. Therefore, future studies should utilize high-fidelity driving simulators or realistic cabin environments to enhance ecological validity. Additionally, while fixation duration and pupil diameter successfully captured macroscopic attentional patterns, future studies should employ fine-grained gaze metrics (e.g., saccadic amplitudes) and tightly controlled luminance conditions to better isolate top-down cognitive workload from exploratory interest, optical (gloss) confounds, and bottom-up pattern-evoked pupillary responses. Finally, given the modest sample size, our correlation analyses provide initial insights; future research utilizing larger cohorts and more comprehensive, multidimensional scales is necessary to robustly decouple interrelated constructs, such as visual complexity, tactile comfort, and subjective preference.

## Figures and Tables

**Table 1:** Objective physical properties of the three surface coatings.

Texture	Roughness Ra (μm)	Roughness Rz (μm)	Gloss 20° (GU)	Gloss 60° (GU)	Gloss 85° (GU)
Smooth	3.004	17.417	1.0	7.5	13.4
Plain	4.673	23.042	0.1	1.3	2.5
Litchi	11.104	53.401	0.1	1.4	4.9

**Table 2:** Image luminance characteristics of the visual stimuli presented on the LCD monitor.

Texture	Color	Mean Luminance (0–255)	Luminance Contrast (RMS)
Smooth	Red	80.281	18.430
	Brown	101.959	17.883
	Green	88.929	18.180
	Blue	87.745	17.976
Plain	Red	109.556	24.732
	Brown	129.482	21.422
	Green	117.429	23.423
	Blue	115.987	23.301
Litchi	Red	117.506	33.241
	Brown	136.041	29.147
	Green	124.843	31.644
	Blue	123.419	31.556

**Table 3:** Definitions of subjective and objective dependent variables.

Dependent Variable	Definition	Unit	Significance	Reference(s)
Softness	Subjective composite rating of material softness/hardness after touching samples	score	Core psychophysical dimension of tactile perception; measures fundamental judgments of contact comfort	Okamoto et al. [6], Tiest [8]
Pleasantness	Subjective composite rating of pleasantness after observing or touching samples	score	Evaluates the overall sensory emotional valence elicited by surface coating characteristics	Etzi et al. [12]
Visual preference	Subjective rating of visual aesthetic inclination based solely on visual observation	score	Assesses subjects’ aesthetic inclinations and acceptance of the material’s CMF visual appearance	Spence [10], Reber et al. [15]
Total fixation duration	Total duration of gaze dwelling within the Area of Interest (AOI) during sample presentation	s	Reflects subjects’ visual cognitive workload and information decoding difficulty	Guo et al. [22], Just & Carpenter [40]
Mean pupil diameter	Average pupil size of both eyes during sample presentation	mm	Serves as a reliable biological marker for emotional arousal and cognitive engagement under constant illumination	Bradley et al. [41]

**Table 4:** Descriptive statistics of subjective and objective dependent variables across experimental conditions.

Factors	Levels	Softness	Pleasantness	Pupil Diameter (mm)	Total Fixation Duration (s)
Texture	Smooth	5.27 ± 0.17	4.77 ± 0.15	3.72 ± 0.10	5.54 ± 0.16
	Plain	4.82 ± 0.12	4.64 ± 0.11	3.59 ± 0.10	5.51 ± 0.14
	Litchi	4.24 ± 0.15	4.30 ± 0.14	3.52 ± 0.10	5.75 ± 0.13
Color	Red	4.58 ± 0.13	4.40 ± 0.13	3.67 ± 0.10	5.66 ± 0.15
	Brown	4.90 ± 0.11	4.64 ± 0.10	3.57 ± 0.10	5.61 ± 0.13
	Green	4.86 ± 0.13	4.53 ± 0.10	3.57 ± 0.10	5.59 ± 0.15
	Blue	4.77 ± 0.13	4.71 ± 0.12	3.65 ± 0.10	5.55 ± 0.16
Stage	Visual	-	4.36 ± 0.10	3.56 ± 0.10	5.50 ± 0.14
	Tactile	-	4.78 ± 0.10	-	-
	Visuo-Tactile	-	4.58 ± 0.11	3.67 ± 0.11	5.69 ± 0.15

**Table 5:** Repeated measures ANOVA results for subjective perception (Softness and Pleasantness). * *p* < 0.05; ** *p* < 0.01.

Dependent Variable	Source of Variance	*df*	*F*	*p*-Value	ηp^2^
Softness	Texture	1.368	15.140	<0.001 **	0.335
	Color	3	2.728	0.049 *	0.083
	Texture × Color	6	1.029	0.408	0.033
Pleasantness	Stage	2	11.580	<0.001 **	0.278
	Texture	1.578	4.177	0.029 *	0.122
	Color	3	3.206	0.027 *	0.097

## Data Availability

The original contributions presented in this study are included in the article. Further inquiries can be directed to the corresponding authors.
